# The biochemical basis for thermoregulation in heat-producing flowers

**DOI:** 10.1038/srep24830

**Published:** 2016-04-20

**Authors:** Yui Umekawa, Roger S. Seymour, Kikukatsu Ito

**Affiliations:** 1 United Graduate School of Agricultural Science, Iwate University, 3-18-8 Ueda, Morioka, Iwate, 020-8550, Japan; 2 School of Biological Sciences, University of Adelaide, Adelaide, SA 5005, Australia; 3 Cryobiofrontier Research Center, Faculty of Agriculture, Iwate University, 3-18-8 Ueda, Morioka, Iwate 020-8550, Japan

## Abstract

Thermoregulation (homeothermy) in animals involves a complex mechanism involving thermal receptors throughout the body and integration in the hypothalamus that controls shivering and non-shivering thermogenesis. The flowers of some ancient families of seed plants show a similar degree of physiological thermoregulation, but by a different mechanism. Here, we show that respiratory control in homeothermic spadices of skunk cabbage (*Symplocarpus renifolius*) is achieved by rate-determining biochemical reactions in which the overall thermodynamic activation energy exhibits a negative value. Moreover, NADPH production, catalyzed by mitochondrial isocitrate dehydrogenase in a chemically endothermic reaction, plays a role in the pre-equilibrium reaction. We propose that a law of chemical equilibrium known as Le Châtelier’s principle governs the homeothermic control in skunk cabbage.

Physiological temperature regulation occurs typically in birds and mammals when their level of heat production increases as the environmental temperature decreases, such that the body temperature is maintained at a relatively constant level^1^. Since 1972, similar thermoregulation has been demonstrated in the thermogenic flowers of certain plants (e.g. *Philodendron*^2^, *Symplocarpus*^3^, *Nelumbo*^4^,^5^ and *Dracunculus*^6^). Although these thermoregulatory flowers are somewhat homeothermic, the increase in heat production at lower environmental temperatures is not sufficient to maintain floral temperature absolutely constant. For example, core temperatures of sacred lotus flowers decrease 0.9 °C with a decrease in environmental temperature of 10.0 °C^5^. Spadices of skunk cabbage are not as precise, equilibrating at 17.6 °C when air temperature is 5 °C, and at 27.6 °C when the air is 30 °C; thus the rate of respiration increases as floral tissue temperature decreases, reaching a maximum at 15 °C^7^. However, if tissue temperature decreases below 15 °C, respiration abruptly diminishes. We call the temperature of maximum respiration the ‘switching temperature’ because below it the thermostat is switched off^7^,^8^. Above the switching temperature, respiration rate is inversely related to temperature, and in a completely reversible way, which is unexpected in biological systems. Although respiratory pathways mediated by mitochondrial alternative oxidase (AOX) and cytochrome *c* oxidase (COX) have been suggested to play a role in thermogenesis in plants^9^,^10^,^11^, the mechanism causing the inverse relationship is currently unknown. In the present study, we hypothesized that control of respiration in skunk cabbage is achieved through a thermodynamic equilibrium with an overall activation energy that is altered by changes in tissue temperature.

## Results and Discussion

**Figure 1:**
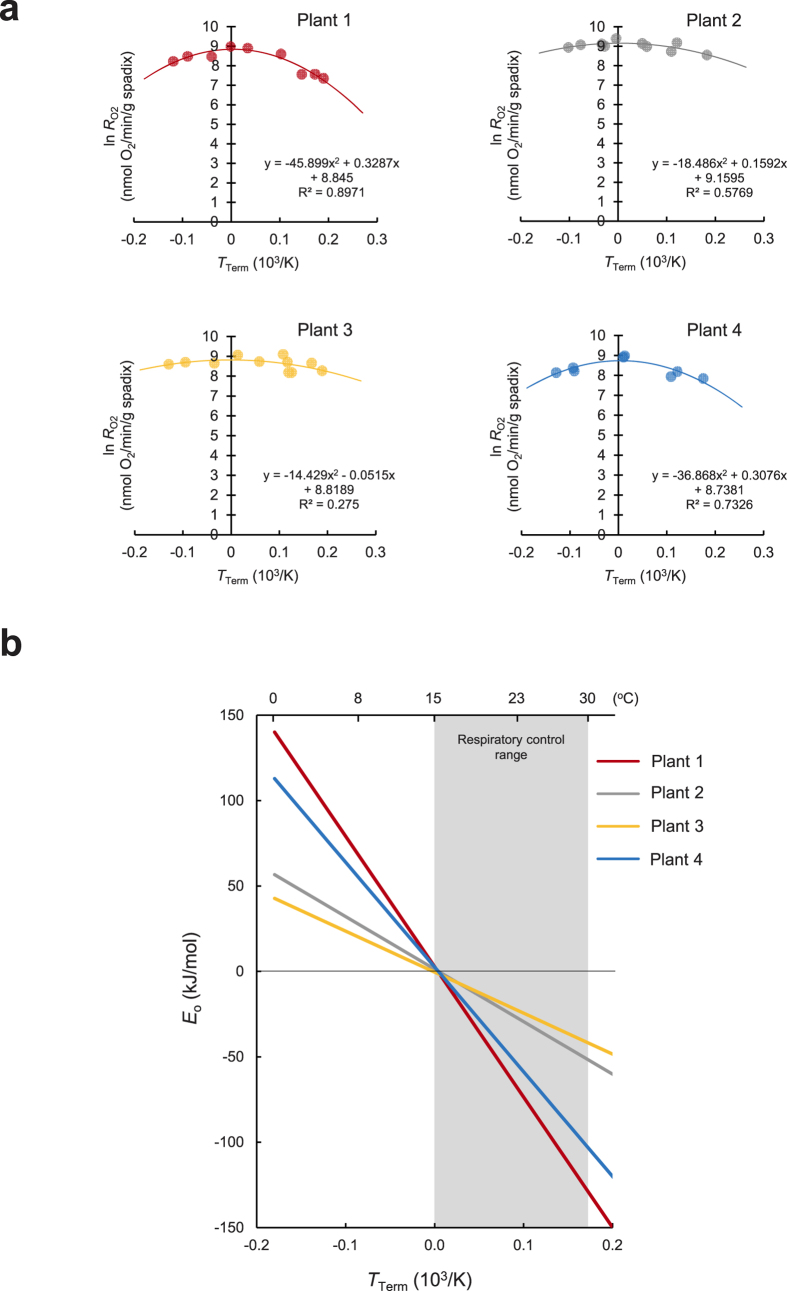
Effects of temperature on changes on respiration of thermogenic spadices of skunk cabbage. (**a**) Curve fitting of individual spadix respiration rate using a modified Arrhenius model. Data are derived from Seymour *et al*.^7^. (**b**) Determination of the relationship between temperature and *E*_o_ for respiration rates in individual spadices. Reference temperature (*T*_REF_) used for analysis is 15 °C.

**Figure 2:**
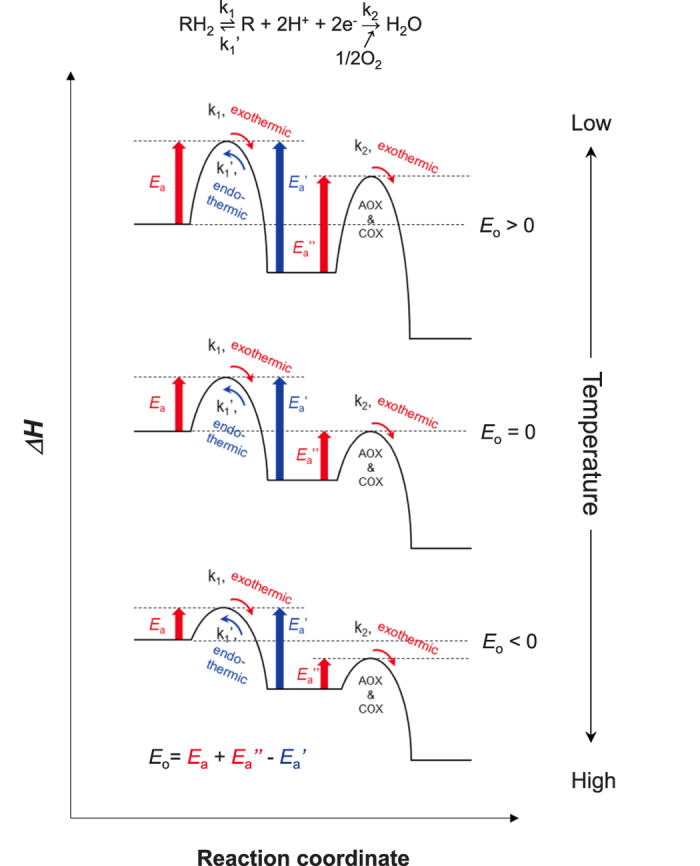
Model of pre-equilibrium reaction in thermogenic spadices of skunk cabbage. The model comprises one leading equilibrium reaction (k_1_ and k_1_′) and one final step of oxygen consumption through mitochondrial terminal oxidases (AOX and COX) (k_2_). The activation energies of each chemical reaction are indicated as follows: an exothermic reaction with reaction constants k_1_ (RH_2_ → R + 2H^+^ + 2e^−^) and k_2_ (1/2 O_2_ + 2H^+^ + 2e^−^ → H_2_O) are expressed as *E*_a_ and *E*_a_^″^, respectively. An endothermic reaction with reaction constant k_1_′ (R + 2H^+^ + 2e^−^ → RH_2_) is indicated as *E*_a_*′*.

To test our hypothesis, we applied the modified Arrhenius model^12^,^13^ to determine the overall activation energy (*E*_o_) for respiration in an intact thermogenic spadix of skunk cabbage. First, we analysed respiration rates obtained through thermal clamping experiments in the field^7^, wherein a range of spadix temperatures were artificially enforced and respiration rate measured at equilibrium. Modified Arrhenius plots provided a better fit of the data than classical Arrhenius plots did (Fig. 1a and Supplementary Figs S1 and S2). Moreover, we could calculate the dynamic changes in *E*_o_ under various temperatures using the ‘switching temperature’ of 15 °C as the reference temperature (*T*_REF_) and other parameters of the model (Fig.1b and Supplementary Table S1). The *E*_o_ values of four independent spadices all decreased as the temperature increased, and there was an apparent intersection point around the switching temperature. Significantly, the *E*_o_ values in the respiration control range were negative, and rates of respiration decreased as temperature increased (Fig. 1b). There is empirical precedence in chemistry for negative activation energies, where an increase in temperature results in a decrease in chemical reaction rates. Examples include the propagation of anionic polymerization^14^ and tryptophan-based, bifunctional, thiourea-catalysed asymmetric Mannich reactions^15^. In contrast, we have found no reports of negative activation energy in biological systems, rather several papers report temperature-dependent changes of *E*_o_ in the range of positive values in plant respiration^16^,^17^,^18^. Importantly, negative activation energy in chemistry implicates a complex chemical reaction containing pre-equilibrium reactions^19^. Namely, a fast reversible step comprises exothermic and endothermic reactions that precede formation of unstable intermediates, and the following rate-limiting reaction determines the entire reaction rate. We propose here that the negative activation energy in homeothermic skunk cabbage could be produced via biochemical pre-equilibrium reactions comprising reversible reactions catalysed by cellular dehydrogenases and a rate-determining reaction catalysed by the mitochondrial terminal oxidases AOX and COX (Fig. 2).

In our model, there are three activation energies: two for the reversible steps of the pre-equilibrium reaction (*E*_a_ for the exothermic direction and *E*_a_′ for the endothermic direction) and one for the final exothermic reaction (*E*_a_″). The relative magnitudes of the activation energies determine whether the overall activation energy is positive or negative. In this case, *E*_o_ is expressed as follows:


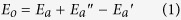


Provided *E*_a_ + *E*_a_″ > *E*_a_′, the activation energy is positive and the rate increases with temperature. However, it is conceivable that *E*_a_ + *E*_a_″ < *E*_a_′, in which case the activation energy is negative and the rate will decrease as temperature increases. This means that the apparent rate of the reverse reaction increases with the temperature and depletes the steady-state concentration of electrons, leading to a decreased respiration rate at higher temperatures.

**Figure 3:**
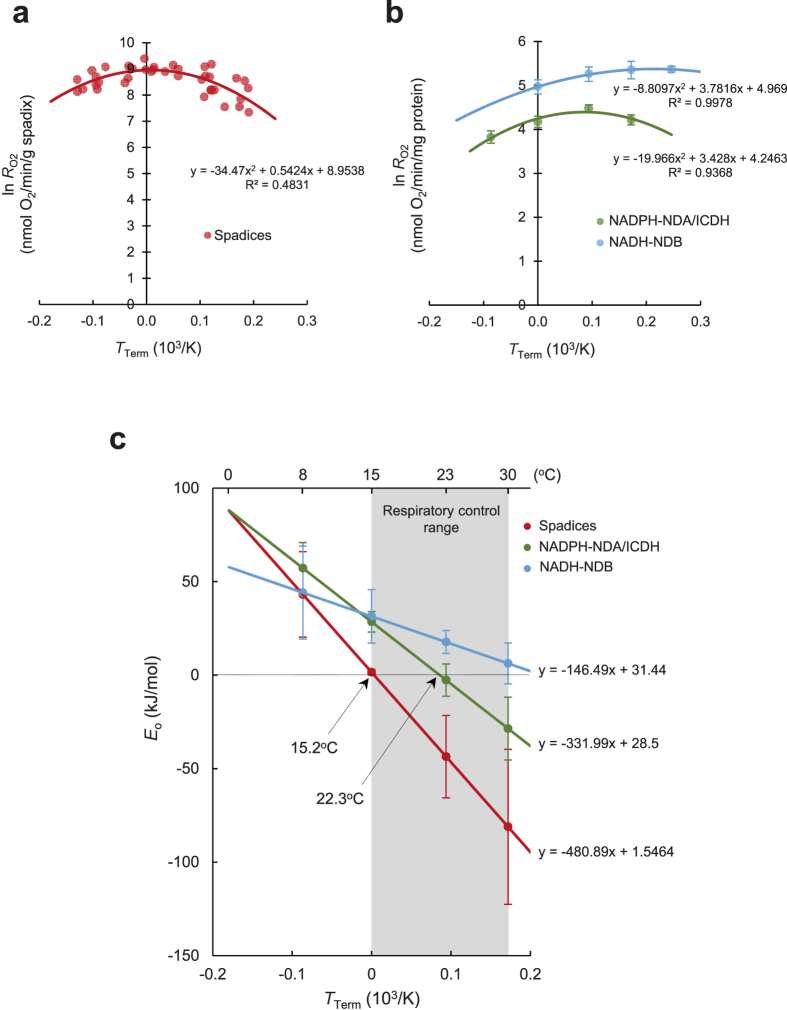
Comparison of the relationship between temperature and *E*_o_ for respiration in intact spadices and mitochondria in skunk cabbage. (**a**) Curve fitting of the respiration rates in intact spadices using a modified Arrhenius model. Data are derived from Seymour *et al*.^7^. (**b**) Curve fitting of the respiration rates in isolated mitochondria using a modified Arrhenius model. Data are from mitochondrial respiration operated by NADP^+^-ICDH and NDA (NADPH-NDA/ICDH; green), and by NAD^+^-NDB (NADH-NDB; light blue). Respiration rates were determined under constant temperature at 8 °C, 15 °C, 23 °C or 30 °C in respiration via NADPH-NDA/ICDH and at 15 °C, 23 °C, 30 °C or 37 °C via NADH-NDB (n = 6). (**c**) Determination of temperature responses of *E*_o_ for intact spadices and mitochondrial respiration. Changes of *E*_o_ value for intact spadices (red), isolated mitochondria (NADPH-NDA/ICDH (green) and NADH-NDB (light blue)) are depicted (n = 6). Intersection points of *E*_o_ are shown for spadices and NADPH-NDA/ICDH at 15.2 °C and 22.3 °C, respectively.

To clarify whether our model for the pre-equilibrium reaction is functional in isolated skunk cabbage mitochondria, we performed *in vitro* respiratory analyses at four different temperatures (in a range from 8 °C to 37 °C), and compared the changes in *E*_o_ with the changes in *E*_o_ in intact spadices (Fig. 3). Because citrate is one of the most abundant organic acids in thermoregulatory male tissues of *Dracunculus vulgaris*^20^ and because our analysis with isolated mitochondria showed that NADP^+^-dependent isocitrate dehydrogenase (ICDH) is the major enzyme that catabolizes isocitrate (which was yielded by aconitase using citrate as a substrate; Supplementary Fig. S3), we focused on the pre-equilibrium reaction mediated by ICDH and type-II rotenone-insensitive internal NADPH dehydrogenase (NDA) in the mitochondria (Fig. 3 and Supplementary Fig. S3). A negative respiration control experiment was conducted with type-II rotenone-insensitive external NADH dehydrogenase (NDB). Because there are no dehydrogenases to convert NAD^+^ back to NADH outside the mitochondria in our experimental system, no pre-equilibrium reaction occurred *in vitro*. Ultimately the data fit well to the second-order polynomial equations of the modified Arrhenius model (Fig. 3a,b). NDB-mediated oxygen consumption using NADH as a substrate never revealed negative activation energy. In contrast, NADPH-NDA/ICDH-mediated oxygen consumption did exhibit negative activation energy, although the temperature at which *E*_o_ was zero (22.3 °C) was higher than it was in intact spadices (15.2 °C; Fig. 3c). Because enzymatic activity for NADP^+^-ICDH increased with the temperature (Q_10_ = 2.0; Supplementary Fig. S3b), a reverse endothermic reaction yielding NADPH would be stimulated at a higher temperature, leading to a shift of the equilibrium to increase the ratio of [NADPH]/[NADP^+^] (Supplementary Fig. S4). It should be noted here that in a new equilibrium, where a higher [NADPH]/[NADP^+^] ratio occurs, the ratio of [ubiquinone (UQ)]/[ubiquinol (UQH_2_)] would be also higher, and, hence, oxygen consumption rates mediated by terminal oxidases would be eventually decreased (Supplementary Fig. S4). These results suggest that the equilibrium shifts with temperature changes to counteract the temperature change and re-establish a new equilibrium. Such behaviour follows a law of chemistry known as Le Châtelier’s principle. It is well-known that this principle has a practical effect only for reactions that are thermodynamically reversible, and our results clearly show that pre-equilibrium reactions containing exothermic and endothermic reversible reactions could be reconstituted *in vitro* using purified mitochondria from thermogenic tissue of skunk cabbage.

The model in Fig. 2 also predicts that temperature-dependent changes of *E*_a_″ determine the temperature at which *E*_o_ becomes zero. Namely, reorganising equation 1, *E*_a_″, the activation energy for AOX- and COX-mediated pathways, is expressed as follows.


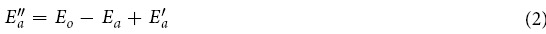


**Figure 4:**
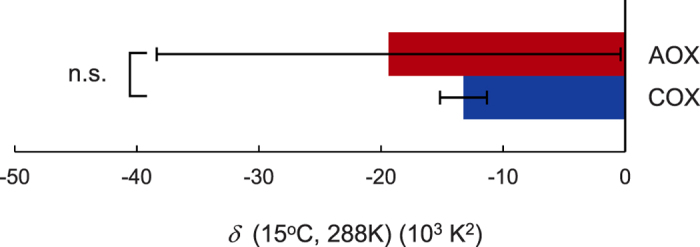
Comparison of the dynamic temperature response (*δ*) of *E*_o_ in mitochondrial respiration mediated by the AOX- and COX- pathways. Values for *δ* of AOX-and COX-mediated oxygen consumptions were analysed (n = 3). n.s.: not significant.

It should be noted here that the value of 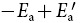 in equation 2 is always identical at any temperature condition, because it shares the same activation energy in the reversible steps of the pre-equilibrium reaction (Fig. 2). Therefore, we attempted to ascertain whether AOX- or COX-mediated pathways contribute equally to setting of the switching temperature. Towards this end, we performed the same *in vitro* mitochondrial respiration assay and measured NADPH-NDA/ICDH-mediated oxygen consumption in the presence of specific inhibitors (KCN for the AOX-pathway and *n*-propyl gallate for the COX-pathway) (Supplementary Fig. 5). Data from both experiments again fit well to the modified Arrhenius model (Supplementary Fig. 5a), which calculated an *E*_o_ of zero for AOX and COX-pathways at 16.1 °C and 24.9 °C, respectively (Supplementary Fig. 5b). These results prompt us to suggest that the AOX-mediated respiration pathway has the greatest influence on the switching temperature in the intact spadices. Further analysis of the dynamic response of *E*_o_ to temperature (*δ*) showed no statistically significant difference between the AOX- and COX-mediated respiratory pathways, which is caused by a larger variation in the data of AOX-mediated respiration (Fig. 4).

**Figure 5:**
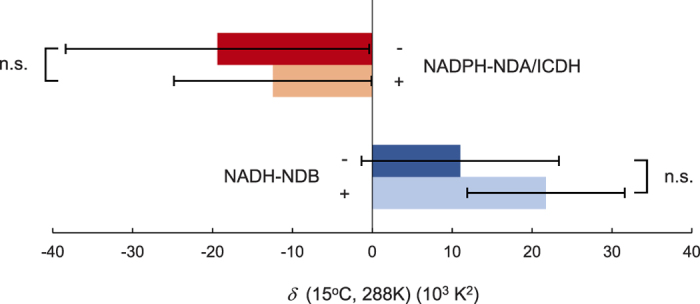
Effects of pyruvate on the dynamic temperature response (*δ*) of *E*_o_ in isolated mitochondria. Values for *δ* of NADPH-NDA/ICDH- and NADH-NDB-mediated oxygen consumptions for AOX capacities were determined in the absence (−) or presence (+) of pyruvate (n = 3). n.s.: not significant.

Finally, we determined the effects of pyruvate on *δ* in AOX-mediated mitochondrial respiration, because pyruvate is known as a positive allosteric modulator of AOX^9^,^21^. In our analysis, AOX capacities in NAPDH-NDA/ICDH- and NADH-NDB-mediated oxygen consumptions showed negative and positive values for *δ*, respectively (Fig. 5). Such an opposite temperature sensitivity suggests a primarily difference of the mechanistic organizations between NAPDH-NDA/ICDH- and NADH-NDB-mediated oxygen consumption rates, although they share the same AOX-mediated respiration pathway from ubiquinol. Importantly, we found no statistically significant effect of pyruvate on *δ* values in each treatment (Fig. 5). Therefore, it is unlikely that activation status of AOX regulates the dynamic temperature response (temperature sensitivity) of *E*_o_ in isolated mitochondria. Alternatively, temperature-dependent equilibrium shifts of the thermodynamically reversible pre-equilibrium reaction, as shown in the present study, may play a role in thermosensation in homeothermic skunk cabbage.

In conclusion, the spadix of skunk cabbage can be considered to act as a chemical reactor, using negative activation energy for its homeothermic regulation. Because overall activation energy for respiration is simply determined by the relative magnitudes of the activation energies of the pre-equilibrium reaction, our proposed model would be robust enough to accommodate large differences in the shape and position of the respiration-tissue temperature curves in other thermoregulatory flowers that have different switching temperatures^7^. More generally, effects of temperature on other cellular biochemical equilibria may follow Le Châtelier’s principle, not only plant thermogenesis but also other biological phenomena such as mammalian non-shivering thermogenesis or fever hyperthermia that may use the same mechanism at least in part to maintain thermal homeostasis.

## Methods

### Plant materials

Thermogenic spadices of skunk cabbage were used for thermal clamping and respiration analyses as described previously^7^. For the purification of mitochondria, thermogenic spadices of skunk cabbage were collected from natural populations grown in a wetland situated in Kanegasaki, Iwate prefecture, Japan, from the end of March to the middle of April 2014 and Nishiwaga, Iwate prefecture, Japan, on 26 May 2014. The collected spadices were stored at 4 °C for 6–18 h until mitochondrial purification.

### Analysis of respiration activities by Arrhenius and modified-Arrhenius equations

Respiration rates measured in our previous study^7^ and mitochondrial respiration rates measured in the present study were used for analyses based on the Arrhenius^22^ and modified-Arrhenius models^12^,^13^. The modified Arrhenius model shown below was used for the curve fitting of respiratory data sets, and parameters (ln *R*_REF_, *E*_o_(*T*_REF_) and *δ* (*T*_REF_)) were determined by a second-order polynomial function as follows^12^,^13^.


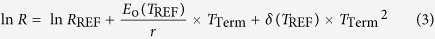


The respiration rate and the activation energy (kJ·mol^−1^) at reference temperature (*T*_REF_) are represented by ln *R*_REF_ and *E*_o_(*T*_REF_), respectively. The temperature sensitivity is represented by *δ* (*T*_REF_) and is equivalent to the slope of the graph showing the relationship between *E*_o_ and temperature, and *r* is the gas constant (8.314 J·K^−1^·mol^−1^). *T*_Term_ represents the temperature term: 
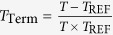
.

The *E*_o_ value is derived from the modified Arrhenius model as follows.


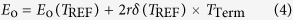


### Mitochondrial purification and respiration analysis

Florets were detached from the spadices and used for the preparation of mitochondria. Purification was performed as previously reported^23^, with the exception that protease inhibitors (cOmplete ULTRA tablets (Roche), or 10 μM E-64 and 0.5 mM 4-(2-Aminoethyl) benzenesulfonyl fluoride hydrochloride) were added upon initiation of the grinding of the florets.

Oxygen consumption rates were determined using freshly prepared mitochondria as described previously^20^,^21^. Mitochondrial respiration with citrate as a substrate was measured in an assay buffer^21^ containing 10 mM citrate, 0.5 mM ADP, 10 mM malonate and 0.1 mM rotenone at 8°C, 15 °C, 23 °C and 30 °C. For respiration assays with NADH as a substrate, 1 mM NADH, 0.5 mM ADP and 0.1 mM rotenone were added to the assay buffer^21^ at 15 °C, 23 °C, 30 °C and 37 °C. Capacities for AOX-mediated pathway were determined as KCN-insensitive (0.5 mM) and *n*-propyl gallate-sensitive (0.1 mM) respiration. Pyruvate (10 mM) was added where indicated. COX capacities were also determined as KCN-sensitive (0.5 mM) and *n*-propyl gallate-insensitive (0.1 mM) respiration. Protein concentrations of purified mitochondria were determined as in our previous report^24^. Mitochondria were stored at −80 °C and used for enzyme assays.

### Enzyme assays

Enzymatic activities for mitochondrial aconitase and NAD^+^- or NADP^+^-ICDH were determined using an Aconitase Assay Kit (Abcam) and an Isocitrate Dehydrogenase Assay Kit (Abcam), respectively, and at 15 °C, 23 °C and 30 °C.

### Statistical analysis

Data were analysed by two-sided Student *t*-test using Microsoft Excel software (2013 for Windows). *P*-values < 0.05 were considered as statistically significant. Results are presented as means ± SD.

## Additional Information

**How to cite this article**: Umekawa, Y. *et al*. The biochemical basis for thermoregulation in heat-producing flowers. *Sci. Rep.* **6**, 24830; doi: 10.1038/srep24830 (2016).

## Supplementary Material

Supplementary Information
