# Supplementary material for: The biochemical basis for thermoregulation in heat-producing flowers
Source: Sci Rep. 2016 Apr 20;6:24830. doi: 10.1038/srep24830 (PMC4837406; doi:10.1038/srep24830)
Supplement: Supplementary Information [file srep24830-s1.pdf]

Supplementary Materials

The biochemical basis for thermoregulation in heat-producing flowers

Yui Umekawa[1], Roger S. Seymour[2], and Kikukatsu Ito[1, 3*]

[1] United Graduate School of Agricultural Science, Iwate University, 3-18-8 Ueda, Morioka, Iwate, 020-8550, Japan.

[2] School of Biological Sciences, University of Adelaide, Adelaide, SA 5005, Australia.

[3] Cryobiofrontier Research Center, Faculty of Agriculture, Iwate University, 3-18-8 Ueda, Morioka, Iwate 020-8550, Japan.

[*]Corresponding author (e-mail: kikuito@iwate-u.ac.jp)

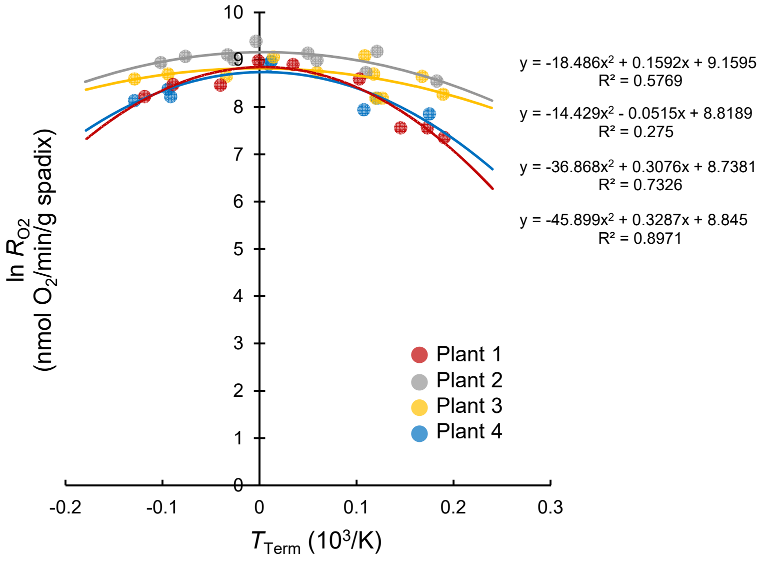

Supplementary Figure S1. Curve fitting of individual spadix respiration rate using a modified Arrhenius model. Individual data in Fig.1a are unified and presented.

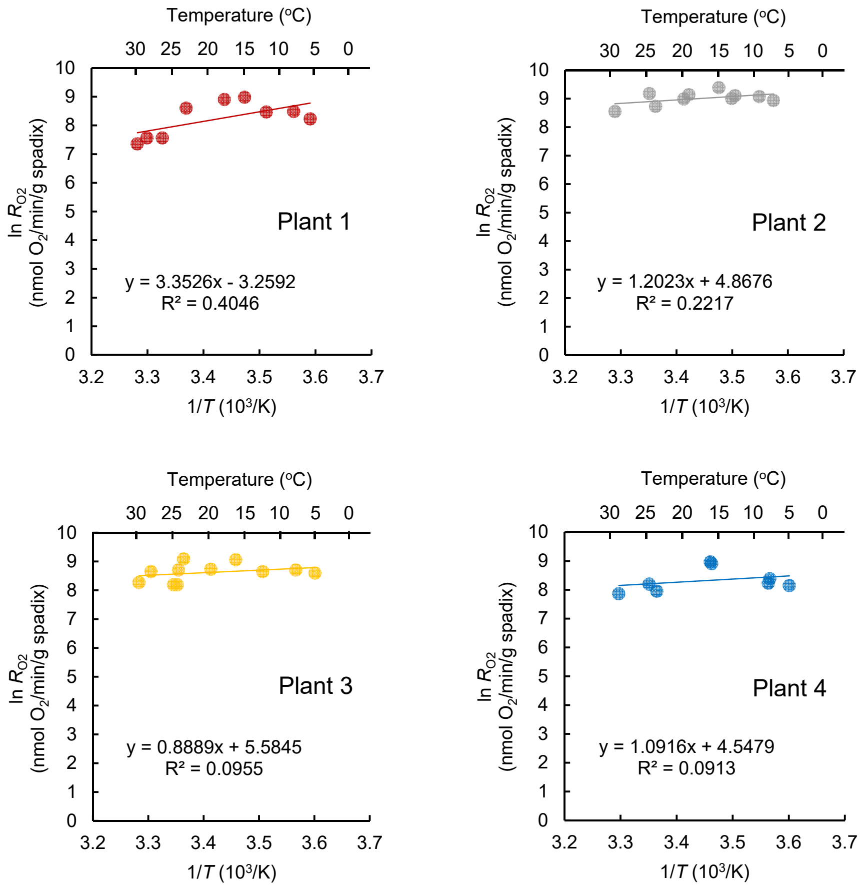

Supplementary Figure S2. Curve fitting of individual spadix respiration rates using a classical Arrhenius model. Data are derived from Seymour *et al.*[1].

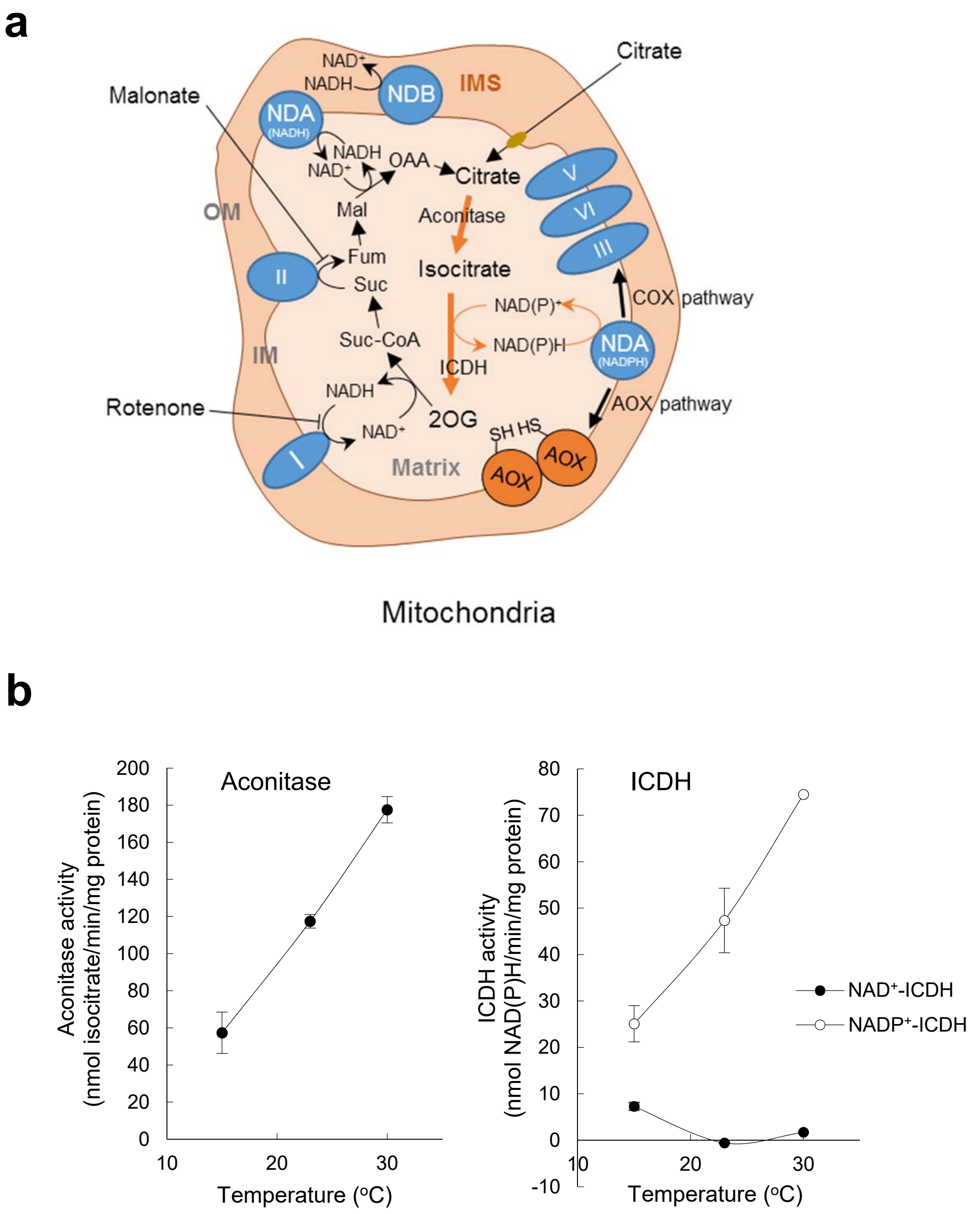

Supplementary Figure S3. Temperature effects on citrate-driven mitochondrial respiration. (**a**) Metabolism and respiration in mitochondria. Citrate is metabolized to 2OG through isocitrate mediated by aconitase and ICDH. In the presence of rotenone and malonate, produced NAD(P)H is oxidized to NAD(P)$^+$ via NDA. Externally added NADH is oxidized by external NADH dehydrogenase NDB. I-V: Complex I-V; ICDH; isocitrate dehydrogenase; 2OG: 2-oxoglutarate; Suc-CoA: Succinyl-CoA; Suc: Succinate; Fum: Fumarate; Mal: Malate; OAA: Oxaloacetate; OM: outer membrane; IM: Inner membrane; IMS: Intermembrane space. (**b**) Temperature response of enzyme activities of aconitase and ICDH (n = 3). There are two types of mitochondrial ICDH: NAD$^+$-type (black) and NADP$^+$-type (white).

$$2NADPH + 2H^{+} + 2\text{-}OG + CO_2 + UQ \underset{k_1'\ (ICDH)}{\overset{k_1\ (NDA)}{\rightleftharpoons}} 2NADP^{+} + Isocitrate + UQH_2 \xrightarrow[\nearrow\ 1/2O_2]{k_2\ (AOX\ \&\ COX)} H_2O$$

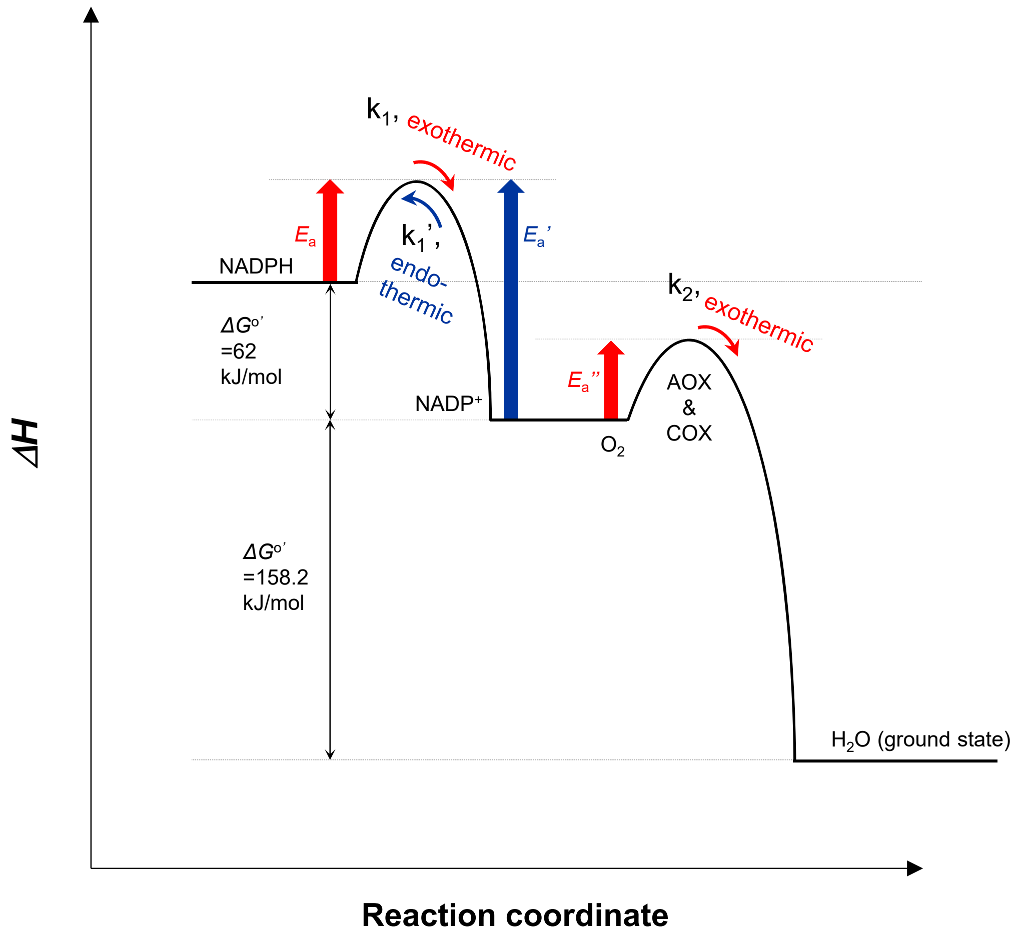

Supplementary Figure S4. A model for pre-equilibrium reaction mediated by mitochondrial NADPH-NDA/ICDH and terminal oxidases (AOX and COX) . Intra-mitochondrially produced NADPH by ICDH show pre-equilibrium reaction by NDA and following oxidation by terminal oxidases (AOX and COX). Rate constants (k_1, k_1' and k_2) and activation energy are depicted as E_a, E_a' and E_a''. Gibbs free energy changes (ΔG^{o}') for each reaction is also shown. 2-OG: 2-oxoglutarate; UQ: Ubiquinone; UQH_2: Ubiquinol.

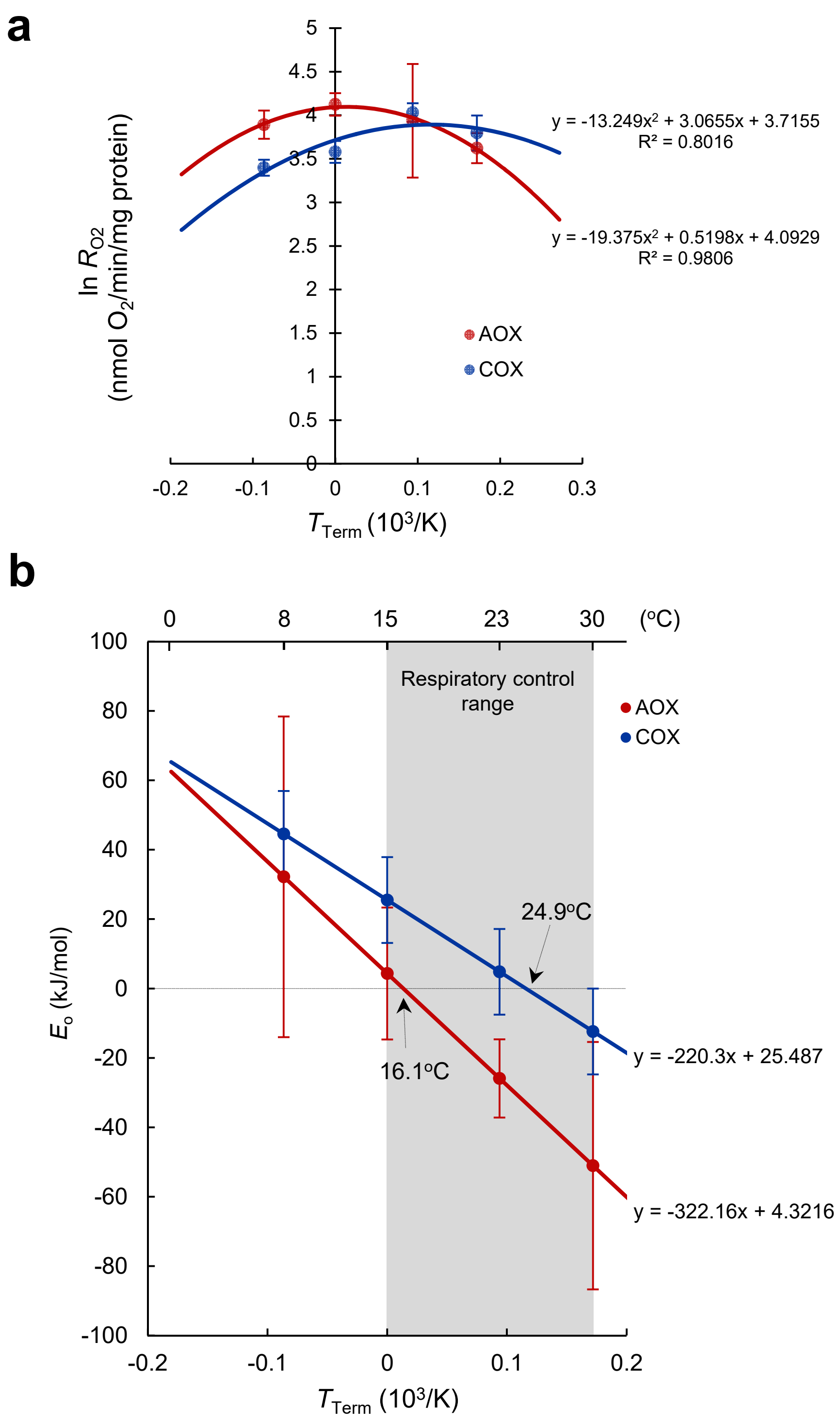

Supplementary Figure S5. Comparison of the relationship between temperature and E_o for mitochondrial respiration mediated by the AOX- and COX-pathways. (**a**) Curve fitting of the mitochondrial respiration via AOX - (red) or COX- (blue) pathway (n = 3). NADPH-NDA/ICDH-mediated oxygen consumption is analysed. (**b**) Determination of the temperature response of E_o for AOX- (red) and COX- (blue) pathways (n = 3). NADPH-NDA/ICDH-mediated oxygen consumption is analysed.

Supplementary Table S1. Respiration variables derived from modified Arrhenius model.

	ln R_{REF} (15°C, 288K) (nmol O_2/min/g spadix)	E_o (15°C, 288K) (kJ/mol)	δ (15°C, 288K) (10^3 K^2)	Temperature at E_o=0 (°C)	Total mass (g)
Plant 1	8.84	2.73	-45.90	15.3	3.35
Plant 2	9.16	1.32	-18.49	15.4	1.81
Plant 3	8.82	-0.43	-14.43	14.9	3.21
Plant 4	8.74	2.56	-36.87	15.4	4.34
Mean ± SD	8.89±0.18	1.55±1.46	-28.92±14.95	15.2±0.20	3.18±1.04

Values for ln R_{REF} (respiration rate), E_o (overall activation energy) and δ (temperature sensitivity) with T_{REF} at 15°C are shown. Temperatures at which E_o shows zero and total mass of each spadix are also indicated.

Supplementary Table S2. Variables of AOX- or COX-mediated respiration pathway.

	ln R_{REF} (15°C, 288K) (nmol O_2/min/mg protein)	E_o (15°C, 288K) (kJ/mol)
AOX	4.09±0.35	4.32±19.01
COX	3.72±0.10	25.49±7.38

NDA-mediated oxygen consumptions for AOX and COX capacities were indicated. Values for ln R_{REF} and E_o with T_{REF} at 15°C were calculated from modified Arrhenius model. Data are means ± SD (n = 3).

Supplementary reference

1. Seymour, R. S., Lindshau, G. & Ito, K. Thermal clamping of temperature-regulating flowers reveals the precision and limits of the biochemical regulatory mechanism. *Planta* **231**, 1291-1300 (2010).
